# Bioinformatics Analysis of *MSH1* Genes of Green Plants: Multiple Parallel Length Expansions, Intron Gains and Losses, Partial Gene Duplications, and Alternative Splicing

**DOI:** 10.3390/ijms241713620

**Published:** 2023-09-03

**Authors:** Ming-Zhu Bai, Yan-Yan Guo

**Affiliations:** College of Plant Protection, Henan Agricultural University, Zhengzhou 450046, China

**Keywords:** *MutS homolog 1* (*MSH1*), bioinformatics analysis, gene expansion, long gene, intron gain and loss, partial gene duplication, transposable element (TE), alternative splicing

## Abstract

*MutS homolog 1* (*MSH1*) is involved in the recombining and repairing of organelle genomes and is essential for maintaining their stability. Previous studies indicated that the length of the gene varied greatly among species and detected species-specific partial gene duplications in *Physcomitrella patens*. However, there are critical gaps in the understanding of the gene size expansion, and the extent of the partial gene duplication of *MSH1* remains unclear. Here, we screened *MSH1* genes in 85 selected species with genome sequences representing the main clades of green plants (Viridiplantae). We identified the *MSH1* gene in all lineages of green plants, except for nine incomplete species, for bioinformatics analysis. The gene is a singleton gene in most of the selected species with conserved amino acids and protein domains. Gene length varies greatly among the species, ranging from 3234 bp in *Ostreococcus tauri* to 805,861 bp in *Cycas panzhihuaensis*. The expansion of *MSH1* repeatedly occurred in multiple clades, especially in Gymnosperms, Orchidaceae, and *Chloranthus spicatus*. *MSH1* has exceptionally long introns in certain species due to the gene length expansion, and the longest intron even reaches 101,025 bp. And the gene length is positively correlated with the proportion of the transposable elements (TEs) in the introns. In addition, gene structure analysis indicated that the *MSH1* of green plants had undergone parallel intron gains and losses in all major lineages. However, the intron number of seed plants (gymnosperm and angiosperm) is relatively stable. All the selected gymnosperms contain 22 introns except for *Gnetum montanum* and *Welwitschia mirabilis*, while all the selected angiosperm species preserve 21 introns except for the ANA grade. Notably, the coding region of *MSH1* in algae presents an exceptionally high GC content (47.7% to 75.5%). Moreover, over one-third of the selected species contain species-specific partial gene duplications of *MSH1*, except for the conserved mosses-specific partial gene duplication. Additionally, we found conserved alternatively spliced *MSH1* transcripts in five species. The study of *MSH1* sheds light on the evolution of the long genes of green plants.

## 1. Introduction

Plant organelle genomes (chloroplast and mitochondria) are derived from endosymbionts with cyanobacteria and α-proteobacterium-like ancestors, respectively [1]. The two genomes encode genes that are essential for photosynthesis and respiration. Furthermore, the plastome and mitogenome evolution of green plants is extremely complex. They present various variations, including size, structure, and gene content [2]. For example, the plastome size ranges from 11,348 bp in *Pilostyles aethiopica* [3] to 242,575 bp in *Pelargonium transvaalense* [4]; Pinaceae and Cupressophytes have lost one copy of the IR (Inverted Repeat) region [5]; the plastome of *Paphiopedilum* has undergone IR expansion and SSC (Small Single Copy) contraction [6]. Moreover, the mitogenome size ranges from 66 kb in *Viscum scurruloideum* [7] to 11.3 Mb in *Silene conica* [8]. Though most of the sequenced mitogenomes had a single ring, a series of lineages found non-canonical mitogenome structures [9]. For example, all the sequenced mitogenomes of Orchidaceae showed a multichromosomal structure [10,11,12,13]. The mitogenome of *Gastrodia eleta* consisted of 19 contigs with a total length of 1340 kb [10], while the mitogenome of *Paphiopedilum micranthum* consisted of 26 contigs with a total length of 447 kb [13].

Many genes related to DNA repair and homologous recombination regulate the stability of the organellar genomes, such as *MSH1*, *POL1A*, *POL1B*, *RECA2*, *RECA3*, *SSB1*, and *SSB2* [14]. The *MSH1* gene regulates the organelle genome stability and alters the plant phenotype [15,16,17,18,19]. The *MSH1* gene was first cloned from the *Arabidopsis* mutant [19], and the gene contains six conserved domains, three of them (DNA binding domain, ATPase domain, and GIY-YIG domain) including recognizable features, and the C-terminus GIY-YIG domain differentiates *MSH1* from other *MutS* homologs (*MSH2*–*MSH6*) and the *MSH1* of yeast [20]. The disruption of *MSH1* increases the repeat-mediated homeologous recombination in *Arabidopsis thaliana* and *Physcomitrella patens* organelle genomes [21,22], and the gene is required to maintain the low mutation rates of the organelle genomes [23]. The mutant of *MSH1* impacts plant growth and induces phenotypic defects, such as variegation, variable growth rate, and delayed maturity [18,24,25,26]. *MSH1* even enhances plant phenotypic plasticity [27]. Moreover, *MSH1* accelerates the sorting of mutations in plant mitochondrial and plastid genomes [15]. All previous studies mainly focused on the function of *MSH1*.

Abdelnoor et al. [20] identified and compared the *MSH1* gene of six plant species, and the gene in these species has 22 exons and 21 introns with canonical splice sites and similar size coding regions. While the gene length ranged from 6.3 kb in *Arabidopsis* to 22 kb in common bean, the extreme length variation provided a unique opportunity to investigate the evolution of long genes. Then, Lin et al. [28] conducted a systematic phylogenetic analysis and inferred that the *MSH1* gene in eukaryotes horizontally transferred from bacteria. Furthermore, Odahara et al. [22] found the partial gene duplication (incomplete gene duplication) of *MSH1* in *P. patens*, and the two copies present a functional differentiation. However, whether the partial gene duplication is species-specific or clade-specific is unknown. Additionally, Wu et al. [23] reconstructed the phylogeny of *MSH1* with sparse sampling, and they found the disjunct distribution of *MSH1* across the tree of life. However, the species of green plants are poorly represented, and there are critical gaps in the extent of the partial gene duplication and the gene size expansion.

Gene length and expression level shape the novelties in the genome. The presence of introns enabled some genes of extraordinary size and the expansion of introns through the insertion of transposable elements (TEs) [29]. Guo et al. [30] defined genes over 20 kb as long genes in *Chloranthus spicatus*. A genome-wide analysis showed that some plant species have exceptionally long genes with a high TE content [30,31]. For instance, the average gene length of *A. thaliana* is 2070 bp (TAIR10), while the average gene length of Chinese pine reaches 25,170 bp [31], which means there is a dynamic evolution of gene length among species. However, the evolution of genes with an extreme length variation in green plants is poorly known. Furthermore, longer genes are less likely to produce duplicates and more likely to exhibit alternative splicing [29], and alternative splicing is widespread in multi-exonic genes [32].

Few studies have combined partial gene duplications, transposable elements, and alternative splicing analyses in gene evolution, and the application of these approaches in the study of gene evolution will expand our knowledge of long genes. Benefiting from the recent progress in sequencing technology, more and more high-quality genomes are available; e.g., more than 1031 genomes representing 788 plant species have been released in the last two decades [33], which provides an excellent opportunity to investigate the evolution of long genes. In this study, we intend to explore the evolution pattern of the *MSH1* gene in green plants. First, we will identify the *MSH1* gene in 85 sequenced genomes covering the main clades of green plants, and we will examine the gene length, gene structure, GC content, splice sites, motif and domain organization, and intron gain and loss; secondly, we will survey the partial gene duplications of *MSH1*; thirdly, we will identify the TEs in the *MSH1* gene and determine the contribution of TEs to the gene length; finally, we will analyze the alternative splicing in the *MSH1* gene based on the annotations and the transcriptome data in the public databases.

## 2. Results

### 2.1. The General Features of MSH1 in the Selected Species

The complete *MSH1* gene in 75 species of green plants was identified, with only transcript sequences available in *Picea abies*, and the other nine species (*Abies alba*, *Ceratopteris richardii*, *Chara braunii*, *Penium margaritaceum*, *Pinus taeda*, *Sequoia sempervirens*, *S. noctiflora*, *Chlorokybus atmophyticus*, and *Monoraphidium neglectum*) with incomplete *MSH1* genes (Table S1). And 33 green plants were newly annotated in this study. The annotations of *A. alba*, *C. richardii*, *P. taeda*, and *S. sempervirens* failed due to a potential *MSH1* gene fragmentation across multiple scaffolds; the annotation of *S. noctiflora* failed due to the absence of exon 19; the annotations of *C. braunii* and *P. margaritaceum* failed due the extremely high GC content; and the annotations of *C. atmophyticus* and *M. neglectum* failed due to the lack complete domains. The nine incomplete sequences were excluded for further analysis. By contrast, we detected no *MSH1* orthologs in four non-green plants, including *Cyanophora paradoxa* of glaucophyte and three species (*Chondrus crispus*, *Galdieria sulphuraria*, and *Cyanidioschyzon merolae*) of Rhodophyta, and the disjunct gene distribution in the outgroup is consistent with Wu et al. [23].

**Figure 1:**
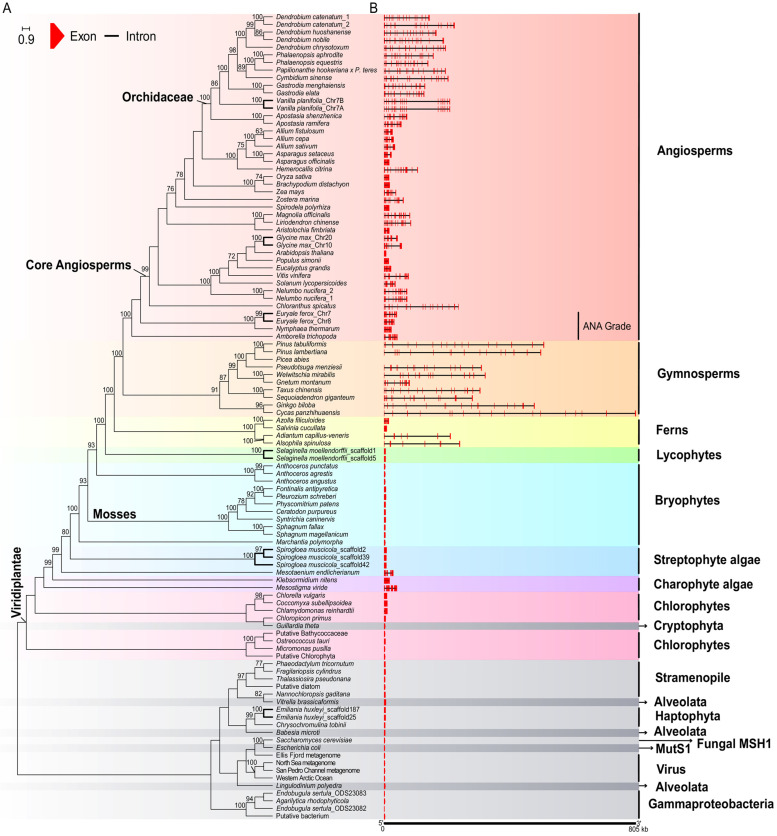
Species phylogeny and gene structures of *MSH1* genes in each species. (**A**) The gene tree was an ML tree constructed in RAxML v8.2.12 based on the coding sequences of *MSH1* in representative green plants, and species names in bold represent non-singleton species; (**B**) The gene structure of *MSH1* were obtained using GSDS 2.0; red boxes represent exons, and black lines represent introns.

We identified the *MSH1* genes of 96 species (76 green plants and 20 non-green plants) in the whole genome data. Sixty of the ninety-six were extracted from the annotations in public databases, while the other 30 were annotated in this study (Table S1). The gene length of green plants ranges from 3234 bp in *Ostreococcus tauri* to 805,861 bp in *Cycas panzhihuaensis*. The coding region of green plants ranges from 1040 amino acids in Putative Chlorophyta to 1584 amino acids in *Mesotigma viride* (Table S1). The gene length of 34 species is over 50 kb, and the gene length of three species is even over 500 kb, while the gene length of non-green species does not exceed 10 kb (Figure 1, Table S1). Expanded *MSH1* genes (over 50 kb) were distributed in multiple clades, including Orchidaceae, *Nelumbo nucifera*, *C. spicatus*, *Liriodendron chinense*, *Magnolia officinalis*, *Vitis vinifera*, *Glycine max*, *Zostera marina*, *Hemerocallis citrina*, gymnosperms, and ferns (*Adiantum capillus-veneris* and *Alsophila spinulosa*) (Table S1). The species in these lineages have large gene sizes and relatively large introns compared with the other selected species, and the gene length positively correlated with the genome size (r = 0.60, *p* < 0.01). The copy number of *MSH1* ranged from one to three; the gene was preserved as a singleton in most of the selected species, except for two copies in *Euryale ferox*, *G. max*, *Selaginella moellendorffii*, *Vanilla planifolia*, and mosses, and three copies in *Spirogloea muscicola* (Table S1). Apart from the relatively low similarity in *E. ferox* (86.7%) and mosses, the sequence similarity of other non-singleton species is over 90%; e.g., the two copies of *V. planifolia* are almost identical, and the similarity of two copies is 97.2% in *S. moellendorffii*.

We identified twenty conserved motifs (Motifs 1–20) in the MSH1 proteins of green plants, and motif composition varies among species. For instance, Orchidaceae, *Asparagus officinalis*, *A. setaceus*, and *Spirodela polyrhiza* contained all of the 20 conserved motifs, while other species lack Motif 14. Furthermore, *G. max* lacks Motif 13 and Motif 18, *Welwitschia mirabilis* lacks Motif 19 and Motif 3, mosses lack Motif 7, and outgroup species contain less conserved motifs (Figure S1). All MSH1 proteins of green plants have the three domains (MutS_I, MutS_V, and GIY-YIG) that were detected in previous studies (Figure S1). According to HMM searches, Motif 4, Motif 11, and Motif 17 encoded the MutS_I domain (70 to 105 aa, 69.9% identity), Motif 1, Motif 6, Motif 12, and Motif 18 encoded the MutS_V domain (153 to 209 aa, 64.6% identity), and Motif 8 and Motif 13 encoded the GIY-YIG domain (28 to 78 aa, 53.7% identity).

Notably, the GC content and gene length varied greatly among species (Table S1). The GC content of the genes ranges from 32% in *Cymbidium sinense* to 71% in *Micromonas pusilla*, and the GC content of the coding regions ranges from 37.8% in *Z. marina* to 75.5% in *Chlamydomonas reinhardtii*. The GC content of the three codon positions is 46.68% to 79.33%, 38.92% to 56.95%, and 27.92% to 91.52%, respectively (Table S1). The codon usages of the three codon positions in most species follow the order of GC_1_ > GC_2_ > GC_3_. The GC content at the third position (GC_3_) drove the high GC content variation. Remarkably, the third position of most bryophytes (46.74% to 77.77%), streptophyte algae (70.06% to 75.59%), charophyte algae (71.45% to 77.90%), and chlorophytes (44.11% to 91.52%) with exceptionally high GC content strongly diverged among species (Figure S2, Table S1).

After removing the eight intronless and two incomplete *MSH1* genes, we calculated 1479 splice sites of 81 sequences representing 66 species of green plants. The canonical splice sites (GT-AG) account for 94.05% (1391 splice sites), while non-canonical splice sites account for 5.95% (88 splice sites), of which GG-CA is the dominant type of non-canonical splice site, with 23 splice sites accounting for 1.56% (Table S2). The non-canonical splice sites are mainly found in the basal clades of green plants (82 of 88 splice sites); e.g., 19 of the 22 splice sites in *Azolla filiculoides* are non-canonical, and 25 of the 27 splice sites in *Chlorella vulgaris* are non-canonical.

**Figure 2:**
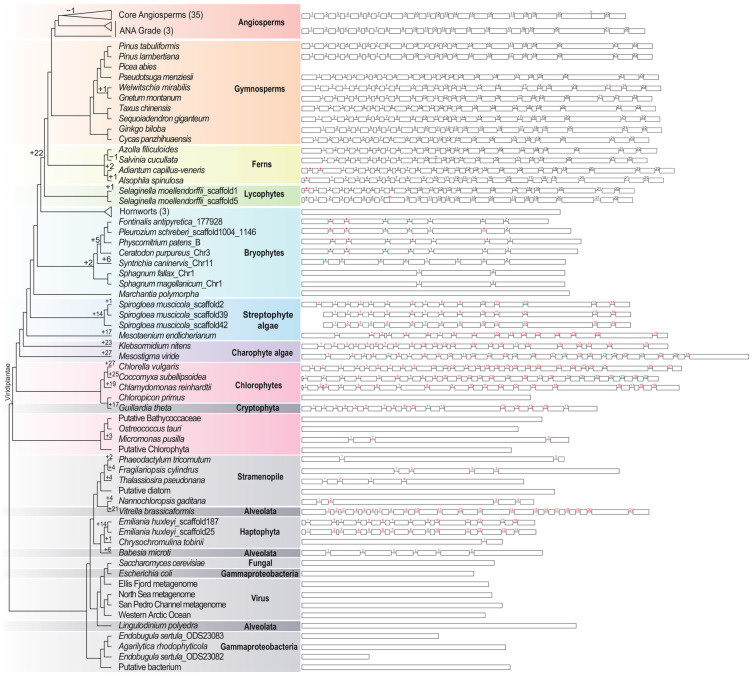
The schematic diagram of the gene structure illustrates the intron gains and losses. The boxes represent exons, and the horizontal lines represent introns; the exon lengths are drawn to scale, and the intron lengths are not drawn to scale. Pluses indicate the number of gained introns, and minuses indicate the number of lost introns. Red numbers and red pluses indicate the position of the newly gained introns.

In addition, the intron number of *MSH1* varied greatly among species, ranging from 0 to 27 introns in *M. viride* and *C. vulgaris* (Table S1). The exon/intron number variation is owing to the intron gains and losses, which occurred multiple times in green plants (Figure 2, Table S1). The intron number in the seed plant is relatively stable. All the selected gymnosperm consisted of 23 exons and 22 introns, except for *Gnetum montanu* and *W. mirabilis*, which consisted of 24 exons and 23 introns. In contrast, all the angiosperms consisted of 22 exons and 21 introns, except for species belonging to the ANA grade (*Amborella trichopoda*, *Nymphaea thermarum*, and *E. ferox*), which consisted of 23 exons and 22 introns (Table S1). The crown clade of the core angiosperms (Mesangiospermae) lost intron 21, while the ancestors of *G. montanu* and *W. mirabilis* gained intron 21′ (Figure 2). We inferred that the ancestors of seed plants consist of 23 exons and 22 introns, and the intron gains and losses of *MSH1* in seed plants are all at the 3′ end of the gene. On the contrary, the basal clades of green plants underwent more frequent intron gains and losses. For example, the intron number of the bryophytes ranged from zero to eight, while the intron number of chlorophytes ranged from 0 to 27. *MSH1* is intronless in *Anthoceros agrestis*, *A. angustus*, *A. punctatus*, *Chloropicon primus*, *Marchantia polymorpha*, Putative Bathycoccaceae, Putative Chlorophyta, and *O. tauri*, and these species are distributed in different clades of the tree. In contrast, the *MSH1* in most other species is intron-rich (Table S1). Notably, most outgroup species (11 of 20) are intronless. Considering the sparse sampling and the poorly resolved species tree, the accurate inference of the intron gains and losses events in the basal group of green plants is unlikely.

### 2.2. Partial Gene Duplications in the MSH1 Gene

**Figure 3:**
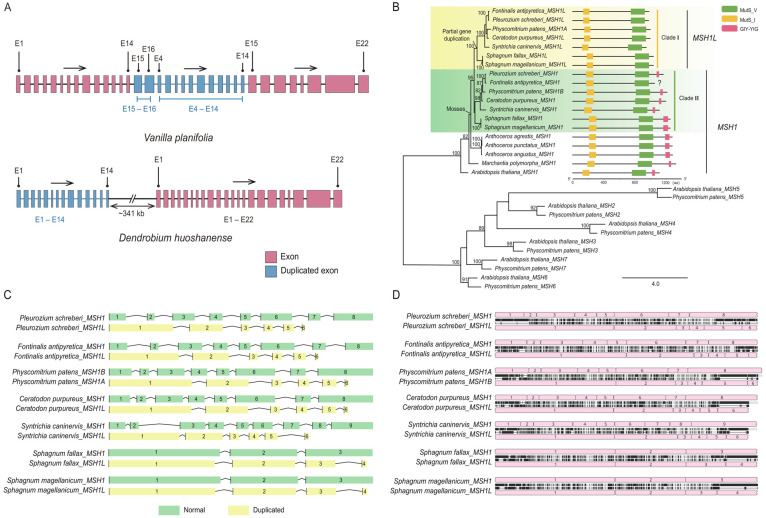
Cases of partial gene duplication detected in this study. (**A**) Diagram of the internal partial gene duplication detected in *Vanilla planifolia* and the external partial gene duplication detected in *Dendrobium huoshanense,* generated in RStudio v4.2.1; (**B**) phylogeny of *MSH1* and *MSH1L* in selected species and other *MSH* genes in *Arabidopsis thaliana* and *Physcomitrella patens,* constructed in RAxML v8.2.12. Clade I of mosses lacking the GIY-YIG domains are the partial gene duplication of *MSH1*, and Clade II of mosses preserve the GIY-YIG domains; (**C**) gene structure comparison of *MSH1* and *MSH1L* in mosses, plotted in RStudio v4.2.1; (**D**) sequence comparison of *MSH1* and *MSH1L* in mosses, obtained from Geneious Prime v2021.2.2. Pink boxes indicate exons; black vertical lines indicate unmatched amino acids.

Notably, all the examined mosses have a lineage-specific partial gene duplicate, and the two copies differ in length and domain. The copy with the GIY-YIG domain is the normal one, and the other copy lacks the GIY-YIG domain derived from partial gene duplication, which was named *MSH1L* in this study (Figure 3). The phylogenetic tree of mosses indicated that all the *MSH1L* in the mosses were grouped into clade I, while all the *MSH1* in the mosses were grouped into clade II; the two copies of the mosses duplicated before the diversification of the mosses, and they correspond to the two copies named *MSH1A* and *MSH1B* in *P. patens* (Figure 3) [22]. Furthermore, we found species-specific partial gene duplication in the other 39 species, such as *Dendrobium huoshanense*, *D. catenatum*, *and H. citrina* (Table S3). Notably, there are 25 seed plants with species-specific partial gene duplications, and the length of the *MSH1* gene in these species is over 20 kb. These partial gene duplications are inserted in the coding regions, or located upstream or downstream of the gene; for example, the duplicated exon 4 to exon 16 in *V. planifolia* is situated in intron 14 of the gene, while the duplicated exon 1 to exon 14 in *D. huoshanense* is located 341 kb upstream of the gene (Table S3, Figure 3).

### 2.3. Transposable Elements in the MSH1 Gene

**Figure 4:**
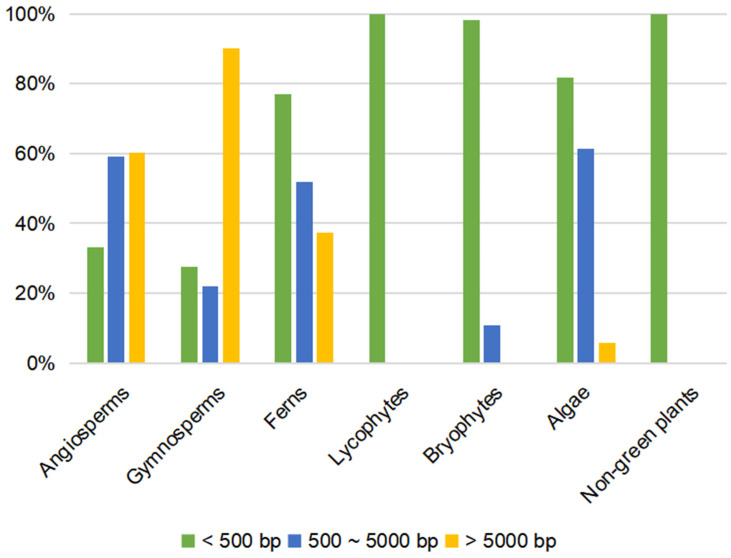
The proportion of different length introns in *MSH1* genes.

**Figure 5:**
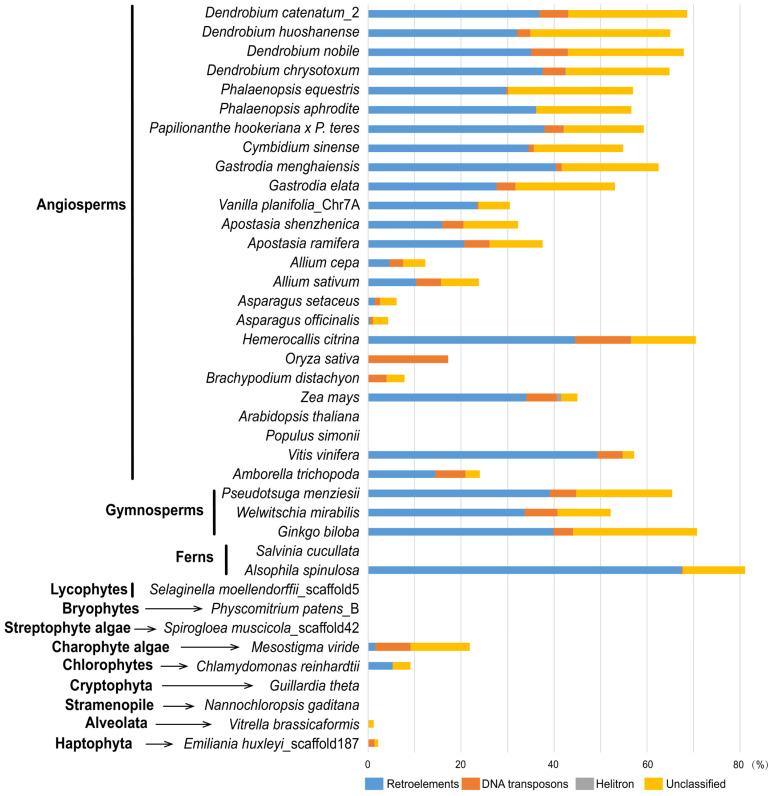
The proportion of the transposable elements of the *MSH1* genes in the 39 representative species.

The intron length ranges from 43 bp in *S. moellendorffii* to 101,025 bp in *V. planifolia* (Table S4). There are 359 introns larger than 5 kb in total, mainly in the seed plants (Figure 4). Interestingly, for Intron 2, Intron 5, Intron 11, Intron 17, and Intron 21 of the seed plants with conserved length, the length of the five introns is shorter than 5 kb in most selected species (Table S4). To further explore the gene expansion of the *MSH1* gene, we counted the TEs of the gene with introns in 39 species representing the main groups (Figure 5). Among them, eight species did not contain transposable elements (*A. thaliana*, *Populus simonii*, *Salvinia cucullata*, *S. moellendorffii*, *P. patens*, *S. muscicola*, *Guillardia theta*, and *Nannochloropsis gaditana*), the TEs ranged from 1.16% (*Vitrella brassicaformis*) to 81.11% (*A. spinulosa*) in the remaining 31 species, and the TEs ranged from 30.49% to 68.59% in Orchidaceae. The gene length positively correlated with the proportion of the TEs (r = 0.81, *p* < 0.01). Among them, 23 species had the highest proportion of retrotransposons, three had the highest proportion of DNA transposons, five had the highest proportion of unclassified transposons, and only three had helitrons. The Gypsy and Copia long terminal repeat (LTR) retrotransposon elements were the dominant components of 20 species (>85%), with a prevalence of Gypsy over the Copia superfamily in 17 species.

### 2.4. Alternative Splicing in the MSH1 Gene

**Figure 6:**
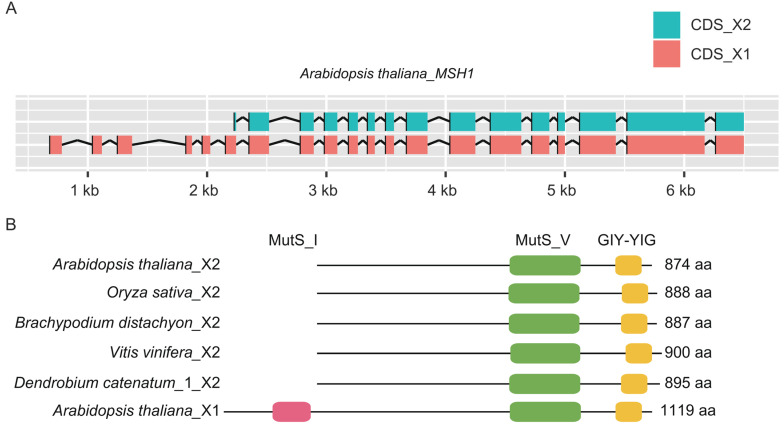
The shared alternative splicing of *MSH1* genes detected in five species. (**A**) Diagram of the shared alternative splicing based on the data of *Arabidopsis,* generated in RStudio v4.2.1; (**B**) diagram of the conserved domains of the shared alternative isoforms, plotted in TBtools v1.098685. X1 represents the constitutive isoform, and X2 represents the shared alternative isoform.

In addition, we found five types of alternative splicing in the nine species, including two alternative acceptor sites, three alternative donor sites, five exon skippings, one mutually exclusive exon, and one other alternative type (Figure S3, Table S5). Besides the constitutive isoform, most of these alternative isoforms are species-specific. We found a shared splice variant in five species (*A. thaliana*, *Brachypodium distachyon*, *D. catenatum*, *Oryza sativa*, and *V. vinifera*). The isoform originated from exon skipping, and starts from 13 bp at the 3′ end of exon 8 and extends to exon 22, with the length ranging from 2622 bp in *A. thaliana* to 2700 bp in *V. vinifera,* and it has lost the MutS_I domain (Figure 6).

### 2.5. The Gene Tree of MSH1

In the gene tree of *MSH1* (Figure 1), the relationship between the main clades is consistent with previous studies [34,35]. However, the inner relationships in most groups are unresolved except for in gymnosperms, and the *G. theta* clusters of green plants with weak support. The copies in the same species cluster together, which means the duplication occurred after the speciation events.

## 3. Discussion

### 3.1. Multiple Parallel Gene Length Expansion of MSH1 in Green Plants

Only six plant species were selected in the previous study [20]. In contrast, we sampled 85 species covering the main clades of green plants and 24 non-green plants. We identified the complete *MSH1* gene in 75 green plants, the transcript sequence in one green plant, and the incomplete *MSH1* gene in the other nine green plants. However, *MSH1* was not detected in four species of Rhodophyta and Glaucophyte, consistent with previous studies [23]. The disjunct distribution of *MSH1* suggests the complex origin of the gene in the ancestors of green plants. The *MSH1* gene is a single-copy gene in most selected species except for the five species with two to three copies. Three species (*E. ferox*, *G. max*, and *S. moellendorffii*) with two copies have undergone paleo-polyploidization events [36,37,38], *V. planifolia* is a phased genome [39], and the three-copy *S. muscicola* experienced a recent whole-genome triplication event [40], suggesting a strong selection for the singleton of the gene in green plants.

The coding region of *MSH1* is relatively conserved in all the selected species (3120 bp to 4752 bp), especially in the seed plants (3330 bp to 3795 bp). However, *MSH1* varied greatly in gene length (3234 bp to 805,861 bp) and intron number (0 to 27) (Table S1). The *MSH1* gene greatly expanded in multiple lineages, especially in the Orchidaceae and Gymnosperms, with the gene length of all the selected species in the two clades over 50 kb. The *MSH1* of Orchidaceae experiences different extents of expansion, with the gene length ranging from 55,035 bp in *Apostasia ramifera* to 225,727 bp in *D. catenatum* (Table S1). All the selected species of gymnosperms have ultra-long *MSH1* genes, ranging from 82,282 bp in *G. montanum* to 805,861 bp in *C. panzhihuaensis*. The most interesting aspect is that the two lineages (Orchidaceae and Gymnosperms) are renowned for their large genome sizes. The genome size of Orchidaceae ranged from 0.33 pg to 55.4 pg [41], and the modal genome size value of the 57 gymnosperm species is 30.0 pg [42]; e.g., the genome size of Chinese pine reaches 25.4 Gb [31]. Moreover, the average intron size of the two clades is much longer than other clades; e.g., the average intron size of *G. elata* is 3252 bp [43], while the average intron size of Chinese pine is 10,034 bp [31]. In contrast, the average intron sizes of *A. thaliana* and *O. sativa* are 161 bp and 469 bp, respectively.

Considering the low length variation of the coding regions, the gene length expansion is mainly induced by the intron size expansion. Most of the introns of gymnosperms and Orchidaceae are over 5000 bp, with 97 introns (48.5%) and 85 introns (26.98%) that are longer than 10 kb, respectively; e.g., the longest intron is the 101,025 bp-long Intron 14 of *V. planifolia* (Table S1). In comparison, all the introns in *Arabidopsis* are shorter than 500 bp. The first intron is the longest in most genes [44]. However, the first intron of *MSH1* is not the longest in most selected species (Table S4). Owing to the extreme length expansion, the gene annotation of *A. alba*, *P. abies*, and *S. sempervirens* failed due to the potential *MSH1* gene being fragmented across multiple scaffolds. Besides the difficulties in assembling, the long genes with multiple introns pose great challenges to gene annotation and identification [31]. Notably, the two samples of *N. nucifera* have similar lengths (73,992 bp and 73,601 bp), while the two samples of *D. catenatum* have distinct sizes (145,440 bp and 225,727 bp) (Table S1). The two samples of *D. catenatum* were sequenced based on different sequencing technologies. The earlier one was sequenced using the Illumina platform [45], while the latter was sequenced based on PacBio long-reads, Illumina short-reads, and Hi-C data [46]. The long repeat sequences in Intron 8 induced the length variation in the two samples. The sequencing platform-induced sequence length variation indicated that the gene length of the short-read sequenced samples might be underestimated, especially the genes with long repeat regions. Moreover, previous studies showed a negative correlation between GC content and intron length, which means that short introns tend to have a higher GC content, while long introns have a lower GC content [47,48]. However, the GC content has no correlation with the intron length in *MSH1*. The GC content of the short introns (<5 kb) ranged from 17.40% to 56.90%, while the GC content of the long introns (≥5 kb) ranged from 27.90% to 56.90%.

Moreover, *MSH1* varied from being intronless in liverworts and hornworts, to having 27 introns in *M. viride* and *C. vulgaris* (Table S1). Notably, intronless, intron-poor, and intron-rich members coappear in the *MSH1* gene, while previous studies found intronless, intron-poor, and intron-rich genes in the same gene family [49]. The intron number variation revealed that the gene had undergone recurrent intron gains and losses (Figure 2). The ancestors of seed plants preserve 23 exons and 22 introns, while the core angiosperms lose one intron, which means that the other 21 introns have existed for over 400 Mya. However, the intron gain and loss events in the basal clades of green plants are more complex, and the coding region of seed-free species exhibited a GC-biased nucleotide composition (Table S1). Furthermore, Gozashti et al. [50] found that intron gains correlated with TEs named Introners; aquatic organisms were 6.5 times more likely to contain Introners than terrestrial organisms, and Introners exist towards insertion into the GC-rich regions.

### 3.2. Partial Gene Duplications in the MSH1 Gene

Notably, we found a lineage-specific partial duplication in the mosses (Figure 2). Based on the sequence comparison and phylogenetic analysis, we inferred that *MSH1L* originated from an ancient partial gene duplication of *MSH1* specific to mosses, and the two copies duplicated before the diversification of mosses, which means the partial duplication copy lasted more than 400 million years in this group. *MSH1L* and *MSH1* correspond to the two *MSH1* genes found in *P. patens* (*MSH1A* and *MSH1B*) (Figure 2) [22]. Odahara et al. [22] found that the function of *MSH1A* on the suppression of organelle recombination is minor, and *MSH1A* might be redundant with *MSH1B*. However, *MSH1L* (also *MSH1A* in *P. patens*) might gain other unknown new functions, which need further verification. Besides the mosses-specific partial gene duplicates, the other partial gene duplicates are all species-specific, and species-specific partial gene duplication tends to appear in long genes (Table S3), which is consistent with previous studies [29]. Furthermore, partial gene duplication followed by neo-functionalization might contribute to the evolutionary innovation reported in other species [51,52]. For example, the species-specific *EXOV* is a partial gene duplicate of *EXOVL* in *A. thaliana*, and the *EXOV* acquired novel direct and indirect interactions with other genes and induced significant morphological effects [52].

### 3.3. The MSH1 Gene Length Is Positively Correlated with the Proportion of Transposable Elements

One of the surprising results of this study was the great length variation of *MSH1*. Apart from the intronless and incomplete genes, the intron content accounts for 4.97% to 99.55% of the gene (Table S4). The extraordinary length of *MSH1* is enabled by the TEs, especially the LTR retrotransposons, and the gene length is positively correlated with the proportion of TEs (R = 0.81, *p* < 0.01). The TEs were also found in the long genes of Chinese pine [31] and *C. spicatus* [30]. TEs insert throughout the genome and contribute to phenotype variation and evolution [53]; e.g., TE insertions at the *FLC* of *Capsella rubella* affect the natural variation in flowering time [54]. On the other hand, TE insertions might regulate the neighbor gene expression; e.g., a TE insertion named *redTE* upstream of the *MdMYB1* is linked to the red skin color of apples [55]. Genes with long introns tend to have a higher expression [30,31]. Furthermore, the suppression of *MSH1* changes the mitogenome conformation [56]. The widespread insertion of TEs in the *MSH1* gene hints at a correlation between TE insertion and organelle genome stability.

### 3.4. Alternative Splicing Detected in the MSH1 Gene

Long introns are associated with high rates of alternative splicing [57]. Notably, five species share a 15-exon alternative isoform in *MSH1*, and the isoform originated from changes in the alternative first exon usage (splicing out the first seven exons and part of exon 8) lacks the MutS_I domain and is relatively shorter than the constitutive splicing (Figure 6). Notably, we found an evolutionarily conserved upstream open reading frames (uORFs) range of 19 aa to 26 aa in the shared alternative isoform. Considering that uORFs potentially regulate stress-related alternative splicing events [32], we inferred that the shared alternative isoform might be stress-related and essential for plant development. Hazra and Mahadani [58] found exon skipping events in *D. officinale* leaves under cold acclimation. Additionally, the alternative splicing analysis indicated that the conventional 5′ splicing sites were not conserved, and generated novel proteins in response to abiotic stress [59,60]. Alternative splicing increases protein versatility and plays a vital role in adaptive evolution, phenotypic novelty, protein diversity, and organism complexity [61,62].

## 4. Materials and Methods

### 4.1. Data Sources

To explore the evolution of the *MSH1* in green plants, we used the genome sequences of 85 species representing the major lineages of green plants, including thirty-nine angiosperms, thirteen gymnosperms, five ferns, one lycophyte, eleven bryophytes, three streptophyte algae, four charophyte algae, and nine chlorophytes (Table S6). We also chose 24 species from the other nine clades as outgroups, following the sampling of Wu et al. [23] (Table S6).

### 4.2. Identification of MSH1 Genes

We downloaded the genome sequences, annotation files, protein sequences, and transcriptome sequences of these species from GenBank or other databases (Table S6). We used BlastP v2.9.0 (E ≤ 1 × 10^−6^) (https://blast.ncbi.nlm.nih.gov/Blast.cgi, accessed on 1 March 2021) to identify the homology of the *MSH1* gene with the protein sequence from *A. thaliana* as a query. Then, the retrieved sequences were used as queries to blast against the species lacking the annotation of the *MSH1* gene. The sequence without annotation was annotated in Geneious Prime v2021.2.2 (Biomatters, Inc., Auckland, NewZealand) and refined manually. Furthermore, we performed a reference assembly in Trinity v2.10.7 [63] to verify the gene annotations. The partial gene duplications were identified via a repeated blast with each exon. Then, we inferred the intron losses and gains of *MSH1* based on parsimony. Considering over half of the species in the outgroup are intronless, we suppose that being intronless is an ancestral form of the gene.

### 4.3. Motif and Gene Structure Analysis

Then, we use HMMER [64] and NCBI-CDD [65] to identify the conserved protein domains. We identified conserved motifs in *MSH1* using MEME version 5.5.4 [66] with the following settings: maximum number of motifs set at 20, and optimum motif width set to ≥6 and ≤100 residues. We visualized the results using TBtools v1.098685 [67]. Finally, we draw the gene structure of *MSH1* using GSDS v2.0 [68]. In addition, the GC content of the three positions was calculated in EMBOSS v6.5.7.0 [69].

### 4.4. Transposable Elements in the MSH1 Genes

We selected 39 species representing all the lineages to characterize the reason for gene expansion. We constructed the species-specific repeat library using RepeatModeler v2.0.2 [70]. Then, we used RepeatMasker v4.1.2 [71] to annotate the TEs in *MSH1* and analyzed the contributions of the four major classes of TEs.

### 4.5. Alternative Splicing in the MSH1 Genes

We selected nine species using the GenBank annotation files and multiple transcripts to analyze alternative splicing. Then, the putative alternative splicing events were identified using AStalavista v4.0 [72] through the GTF file obtained above.

### 4.6. Phylogenetic Analysis

We excluded the copy lacking the GIY-YIG domain in the mosses and the nine incomplete sequences from further analysis, and we included the transcript of *P. abies* for the tree construction. Finally, we preserved 106 sequences representing 96 species (76 green plants and 20 non-green plants) for phylogenetic analysis. The *MSH1* protein-coding sequences alignment was performed using MAFFT v7.407 [73] with the default parameters, and was refined manually. The unalignable regions were removed using Gblock v0.91b [74]. PartitionFinder v2.1.1 [75] was used to determine the optimal partitioning scheme and evolutionary model under the Akaike Information Criterion (AIC). We constructed a maximum likelihood (ML) phylogeny in RAxML v8.2.12 [76] under the GTR+G model with 1000 bootstrap interactions using the inferred alignment of the *MSH1* gene. The species phylogeny obtained was used to infer the intron gains and losses in *MSH1*. Then, we constructed the phylogenetic tree of the mosses using the above methods to clarify the origin of the duplicate copies in the group, and we selected the *MSH1* gene of 12 species and the other *MSH* genes of *A. thaliana* and *P. patens* for the tree construction. The generated trees were visualized using FigTree v1.4.4 (http://tree.bio.ed.ac.uk/software/figtree/, accessed on 11 January 2021).

### 4.7. Statistical Analysis and Visualization

We tested for the correlation of the following two pairs of variables: gene length and genome size, and gene length and the proportion of TEs. The correlation tests were performed using the cor.test function in RStudio v4.2.1 [77], using a Pearson test. The diagrams of the partial gene duplication, alternative splicing, and gene structure comparison of *MSH1* and *MSH1L* in the mosses were plotted in RStudio v4.2.1 [77] with the following packages: GenomicRanges v1.49.0 [78], ggbio v1.46.0 [79], ggplot2 v3.4.1 [80], ggtranscript v0.99.0 [81], and magrittr v2.0.3 [82]. The Figures were arranged and polished in Adobe Illustrator 2020.

## 5. Conclusions

This study provides an overall picture of the evolutionary history of *MSH1* in green plants. We expanded the gene analysis of *MSH1* to 109 sequenced genomes. *MSH1* is universally available in green plants. The gene experienced multiple parallel expansions, intron gains and losses, partial gene duplications, and alternative splicing. Gene length is positively correlated with TEs. Intron gain and loss are mainly reported at the genome scale with distantly related species (e.g., [83]). This study provides a typical example of rampant intron gain and loss in a particular gene with dense sampling, and the intron gains and losses are more complex than expected. The species-specific partial gene duplication in *MSH1* is widespread. However, the accurate annotation is incomplete or lacking, and its function is unknown. Moreover, the mosses-specific partial gene duplication and the alternative splicing shared by five species need further functional verification. In general, partial gene duplication, alternative splicing, and TEs in long introns might lead to neofunctionalization in *MSH1* and boost its adaptation. The expansions of *MSH1* might be potentially correlated to the aberrant mitogenomes and plastomes detected. However, there is no direct link between the *MSH1* gene and the pattern of the organelle genomes. This study suggested that we might underestimate long genes in the plant genome due to the assembling and annotation, and these genes provide unique opportunities to study gene evolution.

## Data Availability

All sequences in this study are openly available from the public database. Datasets for the phylogenetic tree construction are available from the corresponding author.

## Supplementary Materials

The following supporting information can be downloaded at https://www.mdpi.com/article/10.3390/ijms241713620/s1.
