# Supplementary material for: Bioinformatics Analysis of MSH1 Genes of Green Plants: Multiple Parallel Length Expansions, Intron Gains and Losses, Partial Gene Duplications, and Alternative Splicing
Source: Int J Mol Sci. 2023 Sep 3;24(17):13620. doi: 10.3390/ijms241713620 (PMC10487979; doi:10.3390/ijms241713620)
Supplement: Supplementary file 1 [file ijms-24-13620-s001.zip › FigS1-S3.pdf]

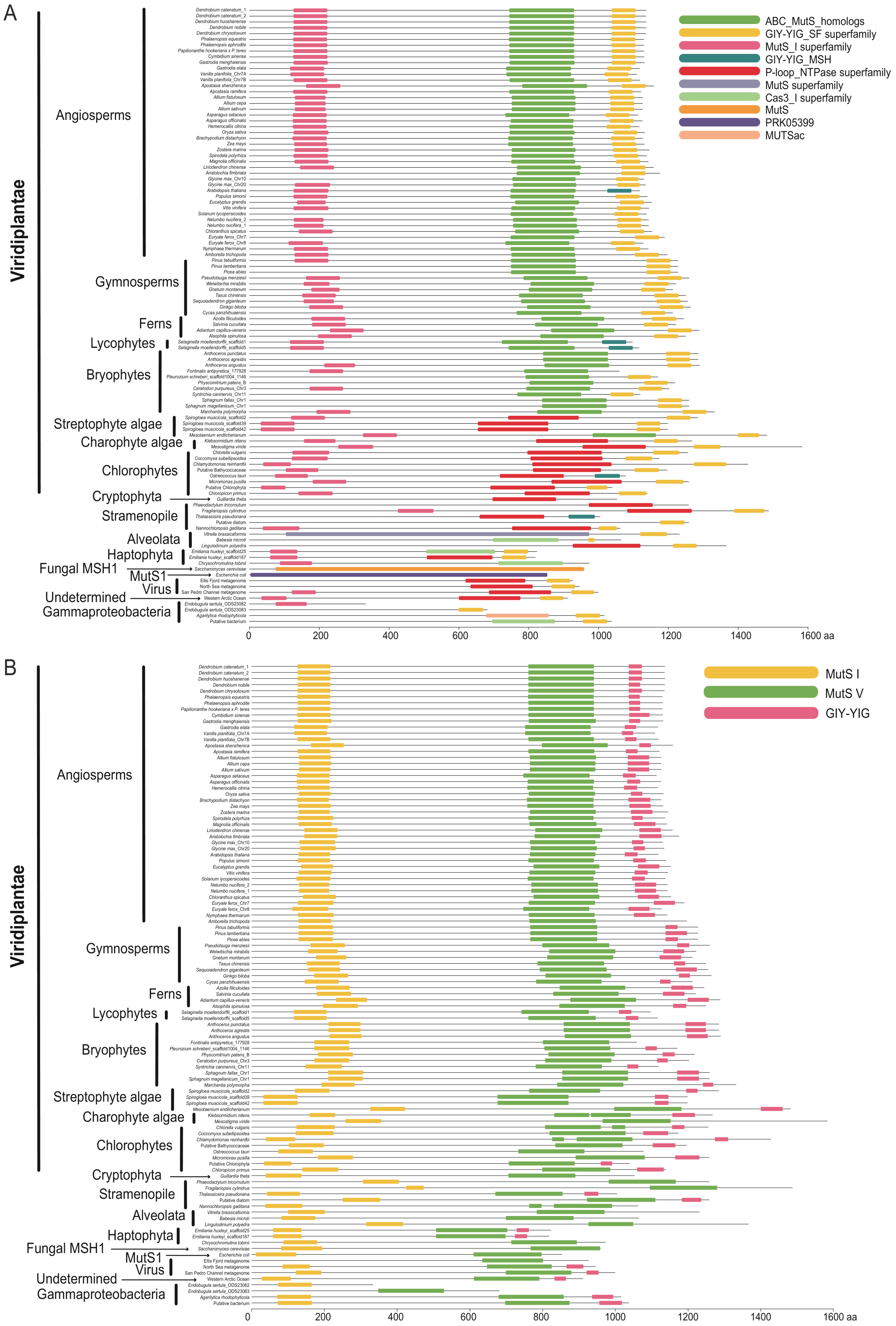
A
Viridiplantae
Angiosperms
Gymnosperms
Ferns
Lycophytes
Bryophytes
Streptophyte algae
Charophyte algae
Chlorophytes
Cryptophyta
Stramenopile
Alveolata
Haptophyta
Fungal MSH1
MutS1
Virus
Undetermined
Gammaproteobacteria
ABC_MutS_homologs
GIY-YIG_SF superfamily
MutS_I superfamily
GIY-YIG_MSH
P-loop_NTPase superfamily
MutS superfamily
Cas3_I superfamily
MutS
PRK05399
MUTSac
0
200
400
600
800
1000
1200
1400
1600 aa
B
Viridiplantae
Angiosperms
Gymnosperms
Ferns
Lycophytes
Bryophytes
Streptophyte algae
Charophyte algae
Chlorophytes
Cryptophyta
Stramenopile
Alveolata
Haptophyta
Fungal MSH1
MutS1
Virus
Undetermined
Gammaproteobacteria
MutS I
MutS V
GIY-YIG
0
200
400
600
800
1000
1200
1400
1600 aa

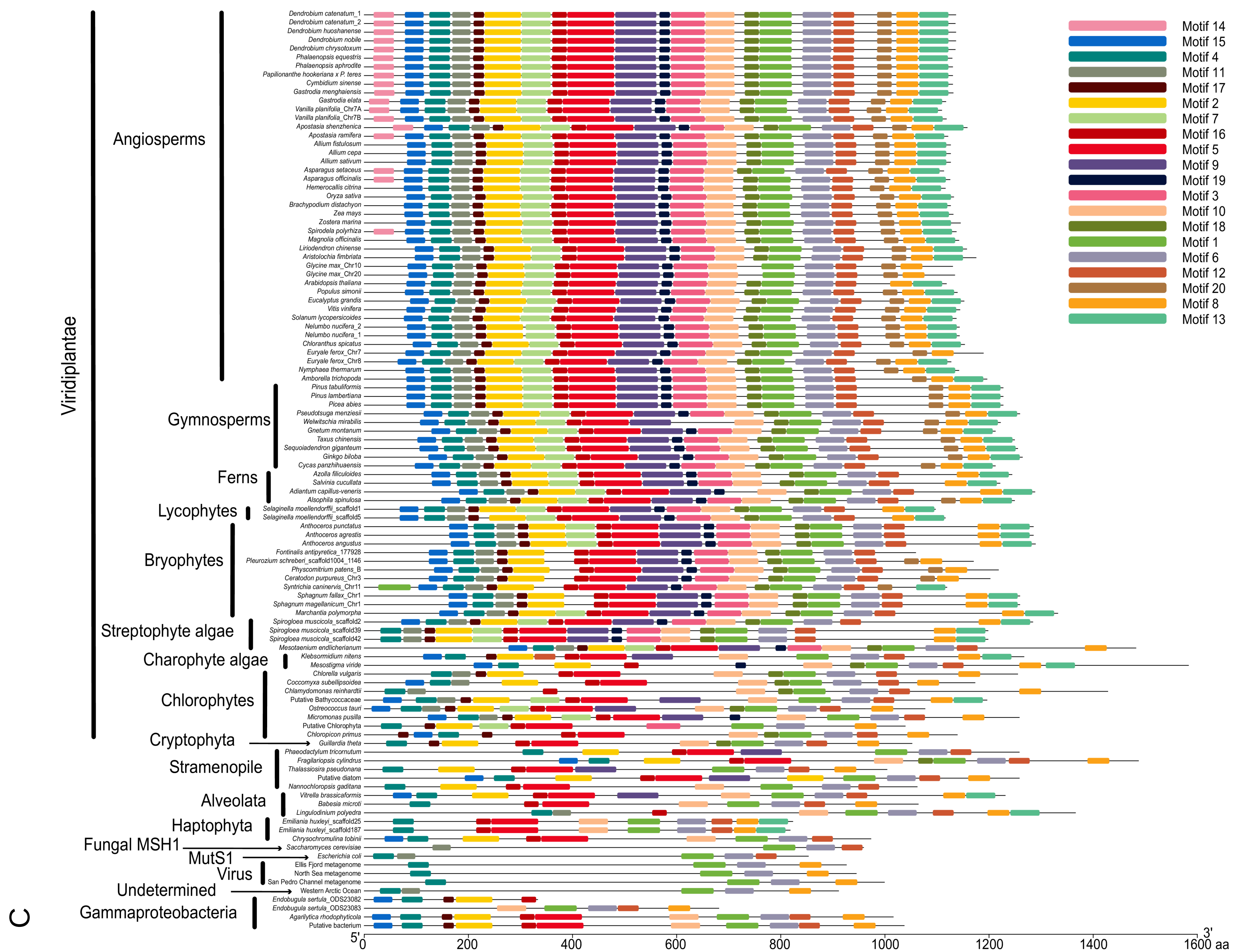
C
Viridiplantae
Angiosperms
Gymnosperms
Ferns
Lycophytes
Bryophytes
Streptophyte algae
Charophyte algae
Chlorophytes
Cryptophyta
Stramenopile
Alveolata
Haptophyta
Fungal MSH1
MutS1
Virus
Undetermined
Gammaproteobacteria
Dendrobium catenatum_1
Dendrobium catenatum_2
Dendrobium huoshanense
Dendrobium nobile
Dendrobium chrysotoxum
Phalaenopsis equestris
Phalaenopsis aphrodite
Papilionanthe hookeriana x P. teres
Cymbidium sinense
Gastrodia menghaiensis
Gastrodia elata
Vanilla planifolia_Chr7A
Vanilla planifolia_Chr7B
Apostasia shenzhenica
Apostasia ramifera
Allium fistulosum
Allium cepa
Allium sativum
Asparagus setaceus
Asparagus officinalis
Hemerocallis citrina
Oryza sativa
Brachypodium distachyon
Zea mays
Zostera marina
Spirodela polyrhiza
Magnolia officinalis
Liriodendron chinense
Aristolochia fimbriata
Glycine max_Chr10
Glycine max_Chr20
Arabidopsis thaliana
Populus simonii
Eucalyptus grandis
Vitis vinifera
Solanum lycopersicoides
Nelumbo nucifera_2
Nelumbo nucifera_1
Chloranthus spicatus
Euryale ferox_Chr7
Euryale ferox_Chr8
Nymphaea thermarum
Amborella trichopoda
Pinus tabuliformis
Pinus lambertiana
Picea abies
Pseudotsuga menziesii
Welwitschia mirabilis
Gnetum montanum
Taxus chinensis
Sequoiadendron giganteum
Ginkgo biloba
Cycas panzhihuaensis
Azolla filiculoides
Salvinia cucullata
Adiantum capillus-veneris
Alsophila spinulosa
Selaginella moellendorffii_scaffold1
Selaginella moellendorffii_scaffold5
Anthoceros punctatus
Anthoceros agrestis
Anthoceros angustus
Fontinalis antipyretica_177928
Pleurozium schreberi_scaffold1004_1146
Physcomitrium patens_B
Ceratodon purpureus_Chr3
Syntrichia caninervis_Chr11
Sphagnum fallax_Chr1
Sphagnum magellanicum_Chr1
Marchantia polymorpha
Spirogloea muscicola_scaffold2
Spirogloea muscicola_scaffold39
Spirogloea muscicola_scaffold42
Mesotaenium endlicherianum
Klebsormidium nitens
Mesostigma viride
Chlorella vulgaris
Coccomyxa subellipsoidea
Chlamydomonas reinhardtii
Putative Bathycoccaceae
Ostreococcus tauri
Micromonas pusilla
Putative Chlorophyta
Chloropicon primus
Guillardia theta
Phaeodactylum tricornutum
Fragilariopsis cylindrus
Thalassiosira pseudonana
Putative diatom
Nannochloropsis gaditana
Vitrella brassicaformis
Babesia microti
Lingulodinium polyedra
Emiliania huxleyi_scaffold25
Emiliania huxleyi_scaffold187
Chrysochromulina tobinii
Saccharomyces cerevisiae
Escherichia coli
Ellis Fjord metagenome
North Sea metagenome
San Pedro Channel metagenome
Western Arctic Ocean
Endobugula sertula_ODS23082
Endobugula sertula_ODS23083
Agarilytica rhodophyticola
Putative bacterium
5'
0
200
400
600
800
1000
1200
1400
1600 aa
3'
Motif 14
Motif 15
Motif 4
Motif 11
Motif 17
Motif 2
Motif 7
Motif 16
Motif 5
Motif 9
Motif 19
Motif 3
Motif 10
Motif 18
Motif 1
Motif 6
Motif 12
Motif 20
Motif 8
Motif 13

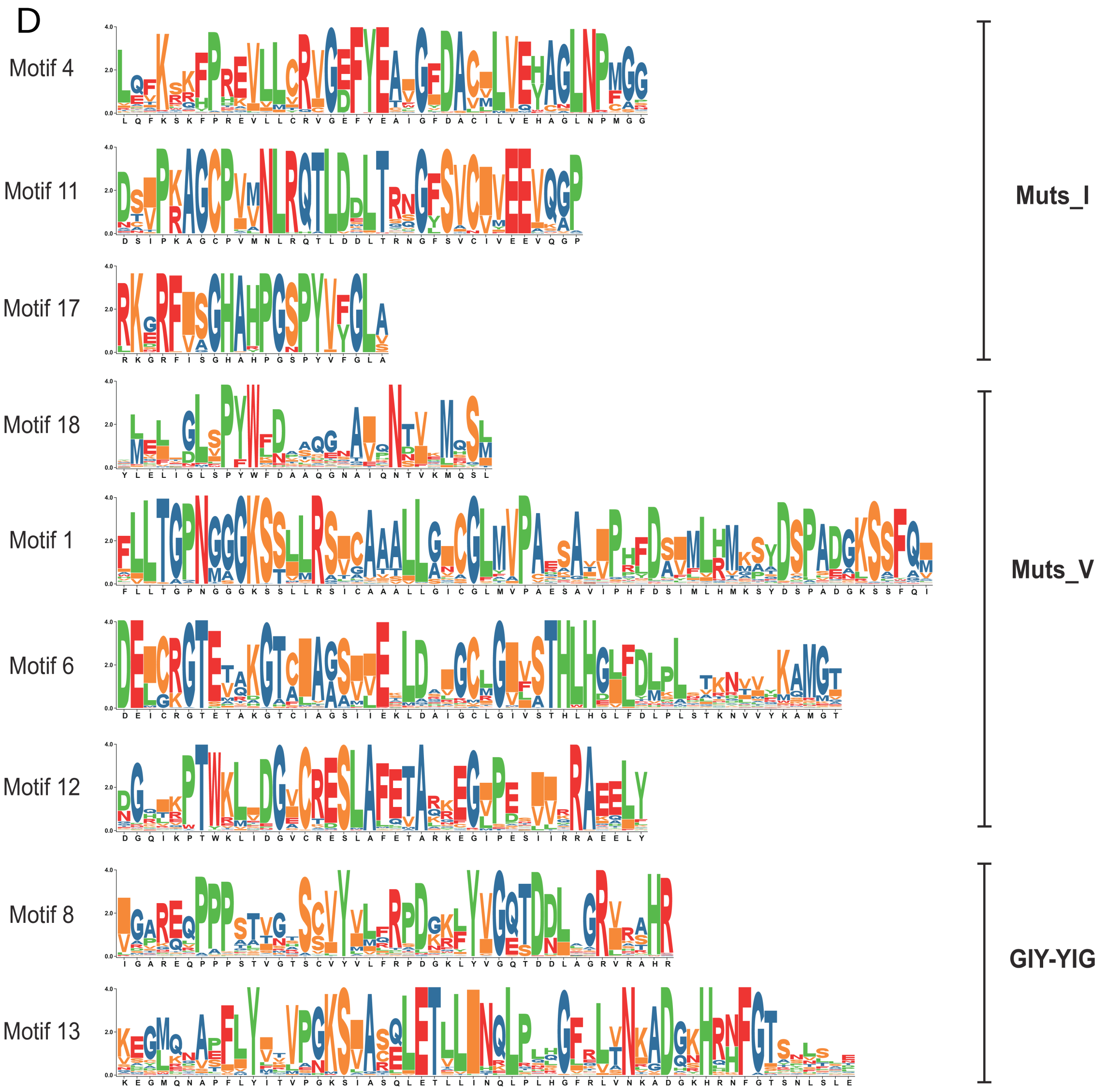

Figure S1. Conserved motifs and domains of *MSH1* genes. (A) The conserved domains of *MSH1* proteins from NCBI CDD; (B) The conserved domains of *MSH1* proteins from HMMER; (C) The conserved motifs of *MSH1* proteins; (D) Sequence logos of the three conserved domains (MutS_I domain, MutS_V domain, and GIY-YIG domain) in *MSH1* proteins.

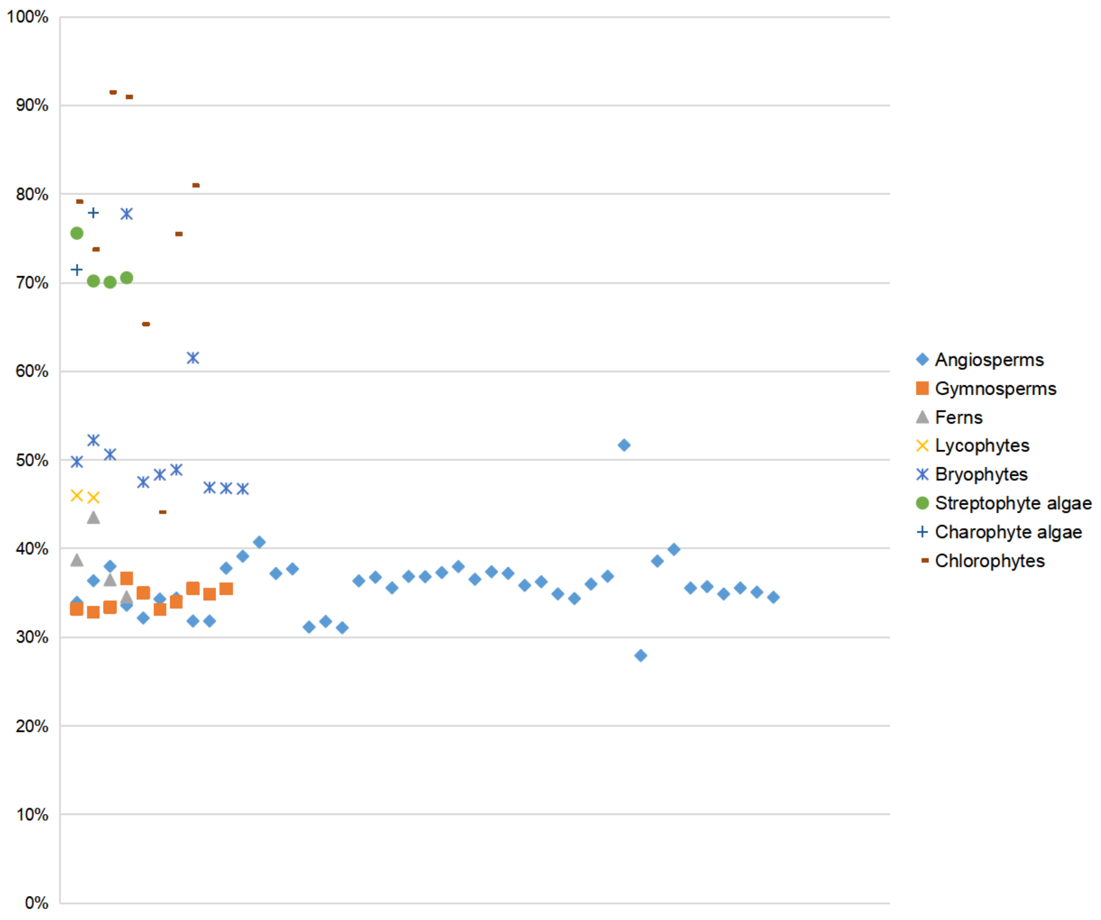

Figure S2. Distribution of GC content of the third codon of *MSH1* gene.

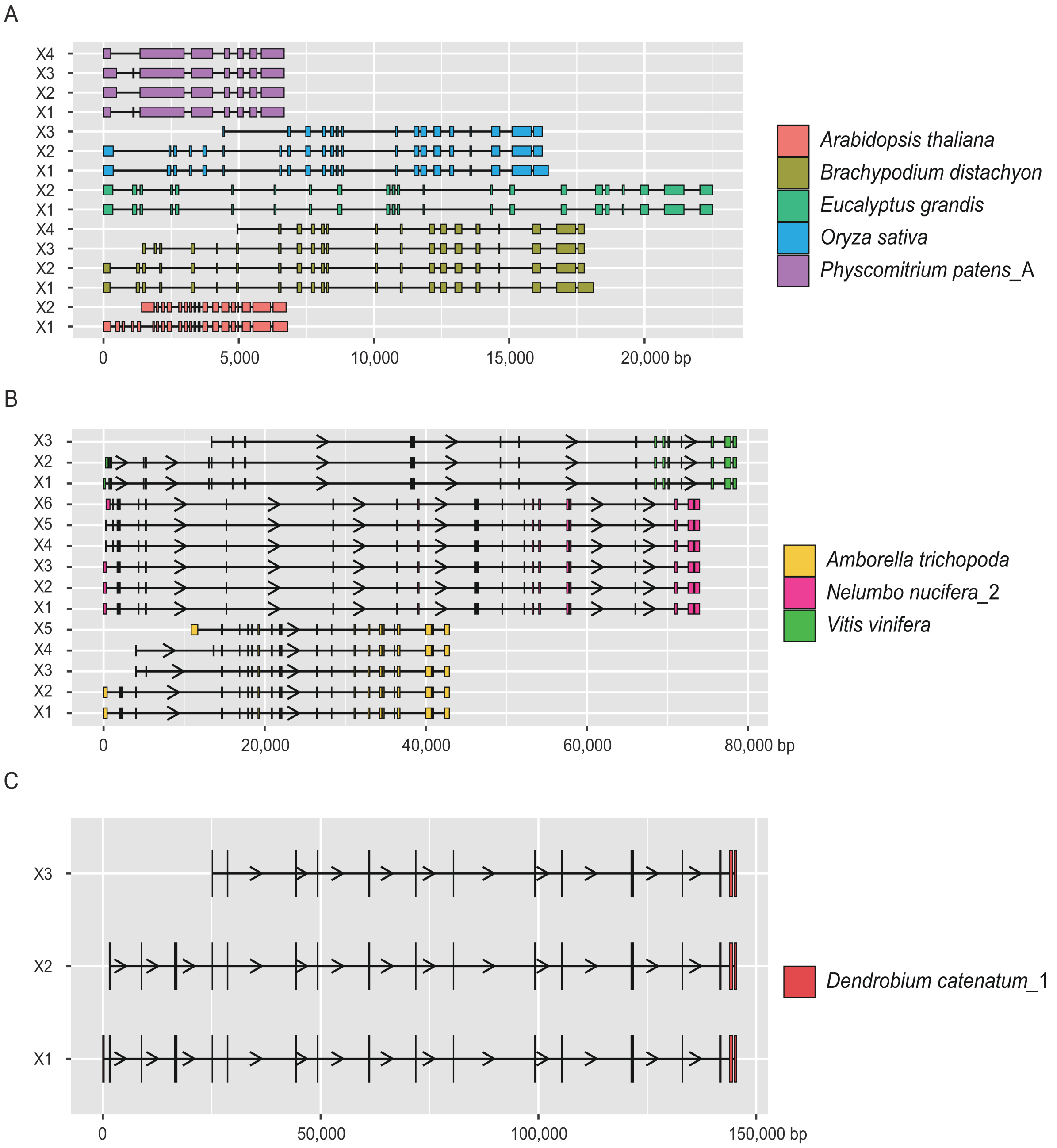

Figure S3. Schematic diagram of different transcripts of the *MSH1* gene in nine species. (A) The transcripts of *Arabidopsis thaliana*, *Brachypodium distachyon*, *Eucalyptus grandis*, *Oryza sativa*, and *Physcomitrella patens*, respectively; (B) The transcripts of *Amborella trichopoda*, *Nelumbo nucifera*, and *Vitis vinifera*, respectively; (C) The transcripts of *Dendrobium catenatum*. Arrows indicate the direction of genes.
